# Key Factors for Implementing Magnetic NDT Method on Thin UHPFRC Bridge Elements

**DOI:** 10.3390/ma14164353

**Published:** 2021-08-04

**Authors:** Sandra Nunes, Mário Pimentel, Aurélio Sine, Paria Mokhberdoran

**Affiliations:** CONSTRUCT-LABEST, Faculty of Engineering (FEUP), University of Porto, 4200-465 Porto, Portugal; mjsp@fe.up.pt (M.P.); aurelio.sine@fe.up.pt (A.S.); pmokhber@fe.up.pt (P.M.)

**Keywords:** ultra-high performance fibre reinforced cementitious composite (UHPFRC), non-destructive method (NDT), magnetic inductance, quality control

## Abstract

This paper provides an overview of the use of the magnetic NDT method for estimating the fibre content, and fibre orientation and efficiency factors in thin UHPFRC elements/layers, along any two orthogonal directions. These parameters are of utmost importance for predicting the post-cracking tensile strength in the directions of interest. After establishing meaningful correlations at the lab-specimen scale, this NDT method can be effectively implemented into quality control protocols at the industrial production scale. The current study critically addresses the influence of key factors associated with using this NDT method in practice and provides recommendations for its efficient implementation.

## 1. Introduction

Ultra-high performance fibre reinforced composites (UHPFRC) are used increasingly for a wide range of structural applications, including innovative solutions for new and existing bridges [1,2,3,4,5]. The economic feasibility and interest of UHPFRC have been demonstrated mainly for new (foot)bridges (structures of extended spans and reduced sections) [1] and in the rehabilitation/strengthening of existing reinforced concrete bridge structures [5]. However, using these advanced materials raises new questions, such as how to control/predict the final fibres distribution in a given structural element, which motivated the development of rational tools to optimise casting processes and fibre distribution control [6,7].

UHPFRC elements can be unreinforced in many circumstances, especially in thin UHPFRC elements such as footbridge deck slabs [3,4,8], watertight jackets or layers over existing concrete elements [5,9], among others. In these cases, the tensile strength of UHPFRC is critical and depends primarily on the properties of the matrix and fibres—such as fibre content, geometry, fibre tensile strength and fibre-to-matrix interface bond—but can also vary significantly according to fibre orientation [10,11]. While the former can be tailored during the mix-design stage, the latter depends on the mould geometry, on the casting procedure, the vibration mode (when applied) and the rheological properties of the material in the fresh state, for example, whether the concrete is self-compacting or not [7]. This means that it is very complicated to define an intrinsic tensile response for UHPFRC, and it is necessary to characterise the representative fibre orientation in the actual structure [11,12]. Non-destructive (NDT) evaluation methods are particularly suited for this purpose.

The most comprehensive method for characterising the distribution and orientation of fibres in a specimen is X-ray tomography [13,14]. However, due to its high cost and limitations in terms of the size of the specimen analysed (which depends on the capacity of the tomograph) it is not currently applicable on the industrial scale. Conversely, the methods based on magnetic induction [15,16,17,18] and electrical resistivity [19,20] are more effective because of their low cost, ease of use, portability and non-destructive nature. The magnetic method consists of generating a local magnetic field and measuring the influence of the fibres on this magnetic field by measuring the inductance. The electrical resistivity method has similarities in its implementation, although it involves measuring a voltage difference between two electrodes. This method is however very sensitive to environmental conditions, mixture proportions and age of testing [20]; for this reason, the magnetic method is preferred to the detriment of the resistivity method.

The authors recently developed a procedure for estimating the post-cracking tensile strength of a thin UHPFRC layer in the directions of interest, eliminating the need for extracting cores or samples from the structure [21]. This procedure is based on magnetic induction [18] and relies on the ferromagnetic properties of the steel fibres for estimating the parameters of the underlying physical model, namely, the fibre content (V_f_), the fibre orientation and efficiency factors [21]. The significance of this NDT method for the quality control of thin UHPFRC elements/layers was demonstrated, and this NDT method is now being used by other research groups [12,22,23].

The performance of the magnetic NDT method on field conditions was assessed during the strengthening of the deck slab of the Chillon Viaducts, in Switzerland, with UHPFRC [9,24]. The main objective of the present work is to critically address the influence of key factors associated with the use of this NDT method in practice and find ways of mitigating their effects on inspection performance. In order to accomplish this objective, a new experimental campaign was carried out aiming to understand the impact of the following key factors: element geometry (thickness and area around the probe), surface condition (roughness of the surface), and fibres segregation (in-depth). Recommendations for the proper implementation of the magnetic NDT method in practice are provided.

To the best of our knowledge, this is the first time that a systematic study has been undertaken on the influence of these key factors for an efficient implementation of the magnetic method, contributing to the advancement of knowledge and promoting its wider use in field applications.

## 2. Non-Destructive Method

### 2.1. Principles

UHPFRC material is constituted by two phases: cementitious matrix and steel fibres. The relative magnetic permeability (Magnetic permeability represents the ease with which a magnetic field is created in an object. The relative magnetic permeability is the ratio between the magnetic permeability of the material and that of the vacuum) of the cementitious matrix equals 1.0 (similar to air) [25], while that of the fibres material (steel) is significantly greater than 1. Therefore, when incorporating short steel fibres in the cementitious matrix, the magnetic permeability of the resulting UHPFRC becomes higher than 1.0.

**Figure 1:**
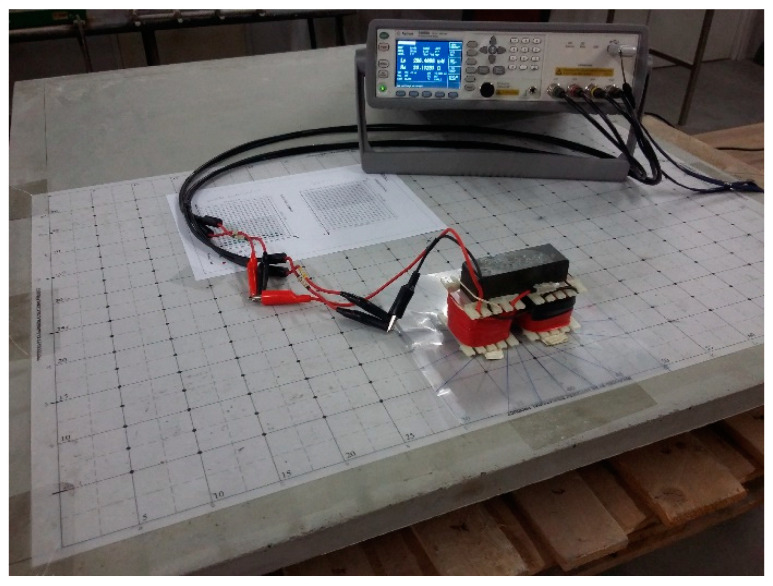
Measurements performed on a UHPFRC large plate using the magnetic probe and an LCR meter.

A magnetic probe was developed for performing inductance measurements [18]. It consists of a U-shaped ferrite core (Siemens ferrite N47) comprising a single copper wire coil around both core legs (see Figure 1). Table 1 summarises the main characteristics of the magnetic probe used in the current study. A high-precision Agilent E4980A LCR Meter (see Figure 1) with a test frequency of 20 Hz and signal level of 2 V (AC) was used to measure both L_UHPFRC_ and L_air_ using a magnetic probe. As shown in Figure 1, it is possible to attach an acetate sheet to the probe legs, with reference lines drawn in different directions, making it easier to align the probe with the direction of interest.

Nunes et al. [18] showed that, for the usual fibre contents, the relative magnetic permeability of UHPFRC (μ_r_) could be readily computed using the following approximation:

(1) $$\mu_{r}\;\approx \;\frac{L_{\mathrm{UHPFRC}}}{L_{\mathrm{air}}}$$

where L_UHPFRC_ is the inductance measured through the UHPFRC material and L_air_ is the inductance measured when the sample is removed (in the air).

L_UHPFRC_, and so the relative permeability of UHPFRC, is governed by the properties of the fibre steel, fibre content (V_f_) and fibre orientation. $\mu_{r}$ increases with V_f_ and changes with the measuring direction (θ_i_) when there is a preferential orientation of the fibres in the UHPFRC layer [18]. When the axis of the magnetic field (direction of probe legs) and the main direction of the fibres are aligned, the measured inductance (and thus $\mu_{r,\;i\;}$) is at its maximum, and vice-versa. As the relative permeability of the cementitious matrix is equal to 1.0, irrespective of its composition and age, $\mu_{r}$ does not change with cementitious matrix composition or age.

### 2.2. Determination of Fibre Content and Fibre Orientation

Nunes et al. [18] proposed the fibre content (μ_r,mean_) and the fibre orientation $\left(\rho_{X}-\rho_{Y}\right)$ indicators for UHPFRC, computed from the inductance measurements in any two orthogonal directions (herein designated as X and Y).

The simplified physical model proposed in [18] was recently generalised, allowing to predict the relative magnetic permeability of a thin UHPFRC layer in any direction and for different types of in-plane fibre distribution functions (herein, we will assume fibres are distributed in XY plane) [26].

#### 2.2.1. Fibre Content

**Figure 2:**
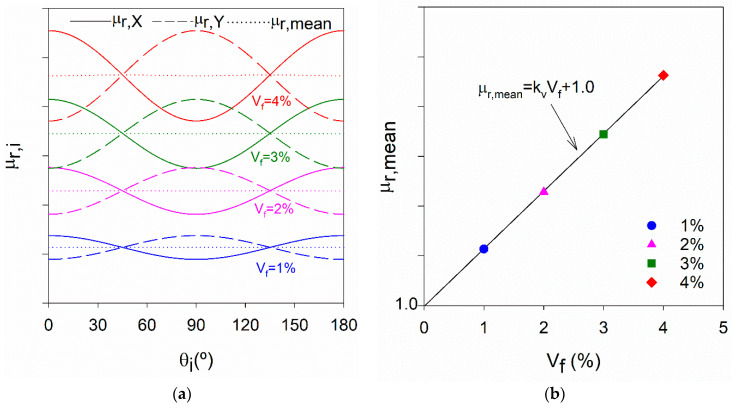
(**a**) Variation of µ_r,i_ and µ_r,mean_ with V_f_ and the measuring direction θ_i_ (preferential fibre orientation coincides with θ_i_ = 0°) (**b**) µ_r,mean_ linear increase with V_f_.

Based on the generalised simplified model [26], it was confirmed that μ_r,mean_ computed from Equation (2) is practically independent of the fibre orientation (Figure 2a) and increases linearly with the fibre content with a proportionality constant $k_{V}$ as depicted in Figure 2b. For V_f_ = 0%, μ_r,mean_ should equal 1.0 (the relative magnetic permeability of the cementitious matrix); therefore, the regression line should intersect the origin. For a given fibre type (or fibres mixture), after calibrating $k_{V}$ by means of representative tests in the laboratory, V_f_ can be estimated using Equation (3).

(2) $$\mu_{r,\mathrm{mean}}=\frac{1}{2}\left(\mu_{r,X}{+\mu }_{r,Y}\right)$$

(3) $$V_{\;f}=\left({\;\mu }_{r,\mathrm{mean}}-1\right){/k}_{V}$$

#### 2.2.2. Fibre Orientation

From the same $\mu_{r,X}$ and $\mu_{r,Y}$ values, the orientation indicator $\left(\rho_{X}-\rho_{Y}\right)$ can be computed from Equation (4), which was shown to be practically independent of the fibre content [18,26]. This indicator allows to identify the direction of preferential orientation of the fibres. Positive or negative $\left(\rho_{X}-\rho_{Y}\right)$ values indicate a preferential orientation along the X and Y directions, respectively. Values of $\left(\rho_{X}-\rho_{Y}\right)$ close to zero indicate a uniform fibre distribution (The only exception is when all fibres are perfectly aligned along a given direction and X/Y measuring directions form a 45° with the preferential orientation direction of the fibres, but this situation rarely occurs in practice, in real applications. This situation can be easily detected by adding a third measuring direction at each measuring point, for example, along the direction forming 45° with X).$\;\left(\rho_{X}-\rho_{Y}\right)$ value also provides a scalar measure of the fibre anisometry with respect to the X- and Y- directions, as described in the following paragraphs.

(4) $${(\rho }_{X}-\rho_{Y})=0.5\frac{\mu_{r,X}-\mu_{r,Y}}{\mu_{r,\mathrm{mean}}-1}$$

**Figure 3:**
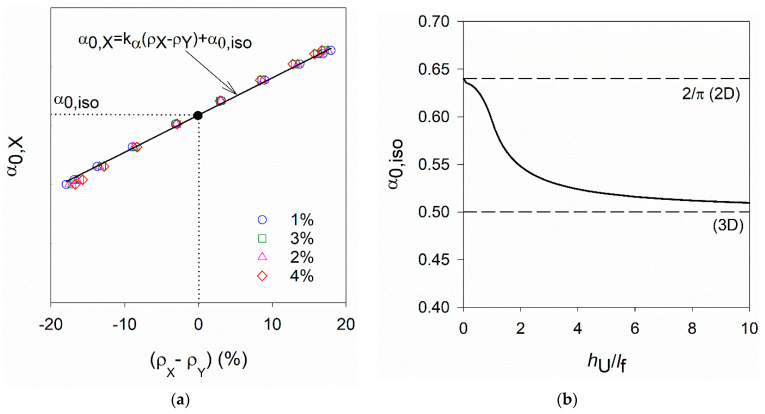
(**a**) Linear increase of α_0,X_ with $\left(\rho_{X}-\rho_{Y}\right);$ (**b**) Variation of α_0,iso_ with h_U_/l_f_ ratio [26].

The fibre orientation factor (α_0_) has been used by several researchers and proved to be an effective tool to characterise the orientation of steel fibres in cracked sections. It was proposed by Krenchel [27] and can be computed as follows:

(5) $$\alpha_{0}{=N}_{f}\;\frac{A_{f}}{V_{f}\;A}$$

where $N_{f}$ is the number of fibres counted in the cross-section, A the cross-section area analysed, $V_{f}$ is the fibre content and $A_{f}$ the cross-section area of one fibre. Based on the generalised simplified model [26], for the most common fibre distribution functions in UHPFRC layers, the relation between the fibre orientation indicator $\left(\rho_{X}-\rho_{Y}\right)$ and the fibre orientation factor along X- and Y- directions ($\alpha_{0,X}$ and $\alpha_{0,Y}$) can be linearly approximated as follows (see Figure 3a).

(6) $$\alpha_{0,X}{=k}_{\alpha }{\;(\rho }_{X}-{\;\rho }_{Y}{)+\alpha }_{0,\mathrm{iso}}\;;\;\alpha_{0,Y}=-k_{\alpha }{\;(\rho }_{X}-{\;\rho }_{Y}{)+\alpha }_{0,\mathrm{iso}}$$

where $k_{\alpha }$ is a constant that needs to be calibrated experimentally for a given fibre type (or fibres mixture), and $\alpha_{0,\mathrm{iso}}$ is the fibre orientation factor corresponding to fibre isometry with respect to X- and Y- directions ($\alpha_{0,X}{=\alpha }_{0,Y}$) [26].

$k_{\alpha }$ can be calibrated based on experimental data obtained, for example, after image analysis of cross-sections normal to X- or Y- directions, using specimens with different fibre contents and a wide range of fibre orientation profiles [21]. $\alpha_{0,\mathrm{iso}}$ can be determined from Figure 3b as a function of the ratio between the UHPFRC element thickness and the fibre length ratio (h_U_/l_f_). When all fibres are lying in the same plane (h_U_/l_f_→0), that is, fibres have a perfect 2D distribution $\alpha_{0,\mathrm{iso}}\approx 0.64$. Otherwise, when fibres have a 3D distribution (h_U_/l_f_→∞), then $\alpha_{0,\mathrm{iso}}\to 0.5$, as shown in Figure 3b.

### 2.3. Estimation of the (Post-Cracking) Tensile Strength

The post-cracking tensile strength of UHPFRC on a given i-direction, f_Utu,i_, is often estimated as

(7) $$f_{\mathrm{Utu},i}{=\tau }_{f}{\;\alpha }_{0,i}{\;\alpha }_{1,i}V_{f}\;\frac{l_{f}}{d_{f}}=\tau_{f}.\lambda_{i}$$

where τ_f_ is the equivalent (rigid-plastic) fibre-to-matrix bond strength and $\lambda_{i}$ is the so-called ‘fibre structure parameter’ [11,21,28], which encompasses fibre parameters related to orientation ($\alpha_{0,i}$), efficiency $\left(\alpha_{1,i}\right)$, content ($V_{f}$) and geometry (l_f_/d_f_). $\lambda_{i}$ can be obtained from the analysis of an image of the cross-section under consideration. This requires cutting several samples from larger specimens and in different directions, carrying a surface treatment (polishing) and, finally, identifying the fibres, which is a time-consuming process and may not be feasible in actual structures. $\lambda_{i}$ can also be determined by X-ray tomography, which allows the fibres orientation and distribution to be determined very precisely. However, it is a much more expensive process and not currently applicable on an industrial scale. Alternatively, Nunes et al. [21] proposed to estimate $\lambda_{i}$ from the NDT measurements. $V_{f}$ and $\alpha_{0,i}$ are estimated using the regression lines presented in Section 2.2, and the fibre efficiency factor ($\alpha_{1,i}$) can be estimated from $\alpha_{0,i}$ using the relation proposed in [11,21].

Previous work by the authors showed that $f_{\mathrm{Utu},i}$ estimates based on $\lambda_{i}$ results obtained from image analysis or the NDT measurements have equivalent accuracy, thereby demonstrating the significance of the proposed NDT method for the quality control of thin UHPFRC elements/layers [21,26].

## 3. Experimental Programme

### 3.1. Materials and Mix-Proportions

The non-proprietary UHPFRC mixes employed in this study are presented in Table 2. The binder phase consisted of ordinary Portland cement (Type I and class 42.5 R), limestone filler (98% CaCO_3_) and silica fume (in suspension with 50% solids content, SiO_2_ > 90%,) with a specific gravity of 3.16, 2.68 and 1.38, respectively. As aggregate, a fine siliceous sand (maximum size of 1 mm) was used with a specific gravity of 2.63 and 0.3% water absorption. Besides the water included in the silica fume, the water amount indicated in Table 2 and a polycarboxylate-based superplasticiser (specific gravity of 1.08 and 40% solid content) were added to the mixture. Concerning the fibres phase, a hybrid mixture of two types of straight steel fibres was used, both having high tensile strength (2100 MPa) and circular cross-section (d_f_ = 0.175 mm), but different slenderness (l_f_/d_f_ = 51 and 69). The performance of this hybrid mixture of fibres was evaluated in a previous experimental campaign that involved performing uniaxial tensile tests on specimens with 1.5% and 3% fibre content, and covering a wide range of orientation distributions [11].

### 3.2. Mixing, Workability and Specimen Preparation

The mixing sequence consisted of the following steps: (1) mixing the binder materials, aggregate and 80% of the mixing water for 2.5 min; (2) stopping to scrape material adhering to the mixing bowl; (3) mixing for another 2.5 min; (4) adding the rest of the water with 75% of the superplasticiser, mixing for 2.5 min and repeating step 2); (5) adding the rest of the superplasticiser; mixing for another 1.5 min; (6) adding the fibres and finally mixing during a further 2 min. In the mixtures with higher fibre contents (3% and 4%), fibres were incorporated in two steps, half the amount at a time.

Immediately after mixing, the mortar flow test was carried out (using a mini-cone according to EFNARC recommendations [29]) to assess the mixture’s flowability by calculating the flow diameter (Dflow) as the mean of two diameters of the spread area.

In order to understand the effect of specimens geometry (thickness and area around the probe), surface condition (roughness of the surface) and fibres segregation (in-depth) on the inductance measurements, and thus relative magnetic permeability, three test series were developed according to Table 3. The geometry, position of the moulds during casting and number of specimens prepared for each test series is indicated in Table 3. In general, special care was taken during the casting of specimens to promote a random orientation of fibres within the specimens. The discharge point was moved randomly along the mould not to let the material flow, preventing a preferential orientation of the fibres. In some specimens of test series C, the orientation of fibres was forced by pouring UHPFRC with the mould placed inside an orientation set-up, where an electromagnetic field was produced, promoting preferential orientation of the fibres, as described in [21]. After casting, the specimens were covered with a plastic sheet and demoulded after one day. Then, the samples were maintained in a chamber under controlled environmental conditions (temperature = 20 ± 2 °C and relative humidity > 95%).

As the relative permeability of the cementitious matrix is equal to 1.0, irrespective of its composition and age, the inductance measurements could be carried out immediately after demolding.

### 3.3. NDT Testing

#### 3.3.1. Test Series A

**Figure 4:**
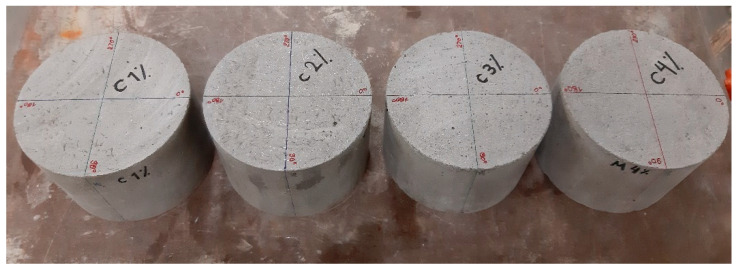
Specimens used in test series A (with original thickness).

**Figure 5:**
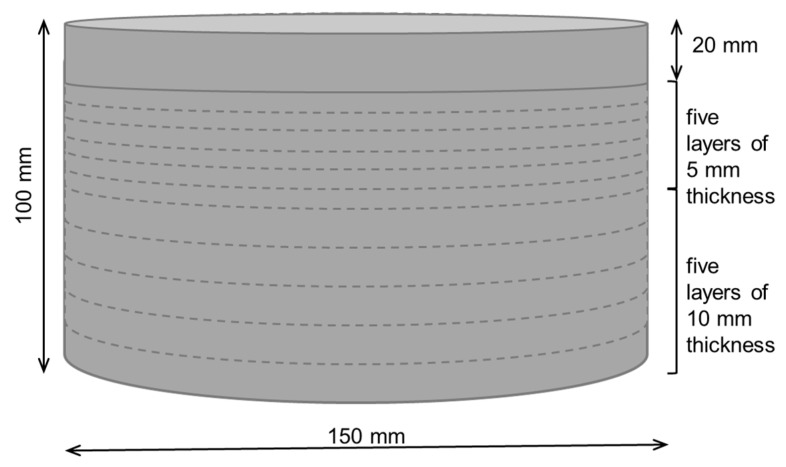
Successive removal of layers of the original cylinder.

In order to assess the effect of the specimen’s thickness on the relative magnetic permeability evaluated using the NDT method, inductance measurements were performed on cylindrical specimens (150 mm diameter) with decreasing thickness. The probe was placed in the centre of the moulded surface of the cylinder and inductance measurements were taken every 15°. The choice of a cylindrical specimen for this series of tests was dictated by the greater ease of handling during the cutting process. The specimens had an original thickness of 100 mm (see Figure 4), from which layers were successively cut (using a diamond blade saw) and removed until the height was reduced to 20 mm. The first five layers to be removed had 10 mm thickness, while the remaining had just about 5 mm (see Figure 5). Inductance measurements, with the probe placed in the centre of the specimen, were repeated at each stage, always using the same surface (moulded face).

#### 3.3.2. Test Series B

**Figure 6:**
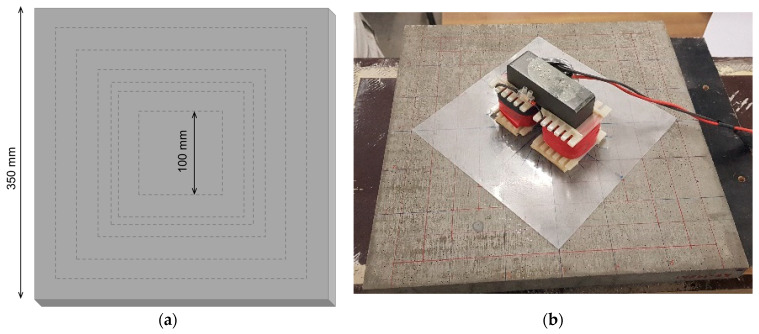
(**a**) Successive reduction of plate size; (**b**) Inductance measurements in the centre of the plate (original size).

In order to assess the effect of specimen area, three large plates of 350 × 350 × 30 mm^3^ were produced. Two of these plates were cast horizontally (here referenced as H1 and H2), while the other was cast in the vertical position (here referenced as V1). The plate size was reduced from 300 mm to 100 mm by cutting it with a diamond blade saw, 10–25 mm at a time (Figure 6a). Inductance measurements were repeated at each stage, with the probe placed in the centre of the plate and always using the same surface (moulded face). Again, inductance measurements were taken every 15° (see Figure 6b).

#### 3.3.3. Test Series C

**Figure 7:**
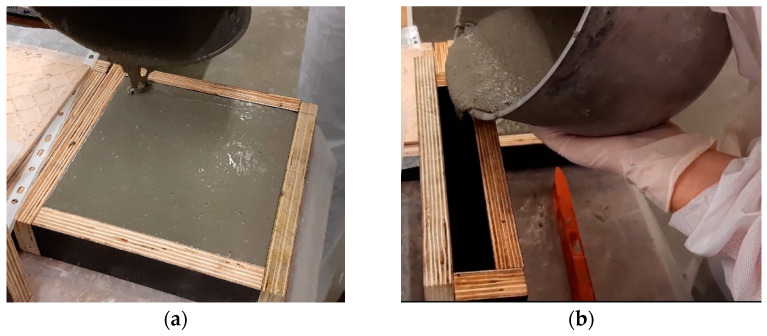
Casting of UHPFRC plates with moulds in (**a**) horizontal position; (**b**) vertical position.

**Figure 8:**
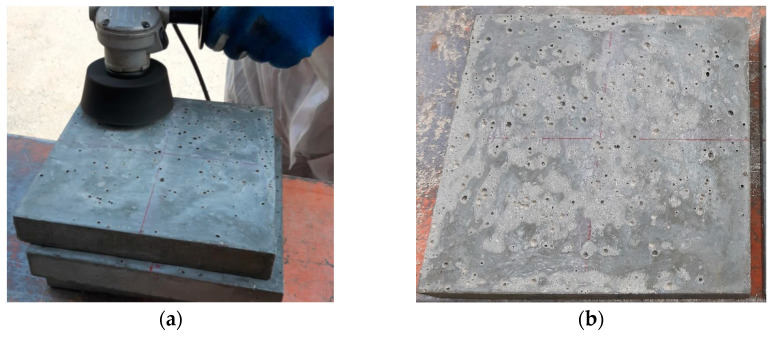
(**a**) Process of polishing the casting surface; (**b**) Surface after polishing.

Within test series C, 12 plate specimens of 200 × 200 × 30 mm^3^ were produced. For each fibre content, two specimens were cast horizontally (one with random and the other with preferential fibre orientation), and one specimen was cast vertically, as shown in Figure 7. Inductance measurements were taken on both surfaces of each specimen: one casting surface and one moulded surface, in the case of horizontal specimens, and two moulded surfaces in vertical specimens. Additionally, a polishing machine was used in the casting surface of horizontal specimens to reduce their roughness (smoothed surface), and measurements were repeated on the casting surfaces after polishing (see Figure 8). The testing conditions and specimen referencing are detailed in Table 4.

For each test condition, the magnetic probe was centred in the plate and rotated by 15° to take the inductance measurements in different directions (θ_i_ = 0° up to 180°), similar to the test series A and B.

## 4. Results and Discussion

### 4.1. Workability

**Figure 9:**
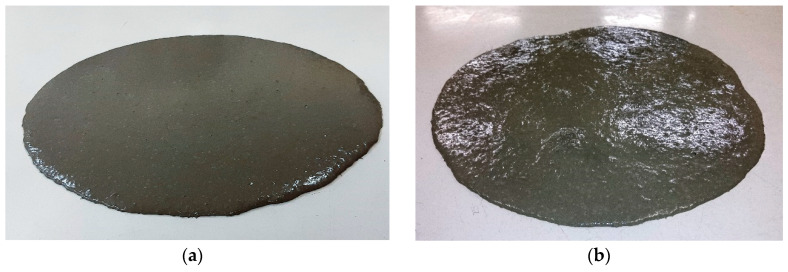
Final spread area of UHPFRC mixes with a fibre content of (**a**) 1% and (**b**) 4%.

Table 5 presents the slump-flow diameter results of different mixes tested at different dates. All tested mixtures are self-compacting, exhibiting good flowability. An increase in fibre content leads to a decrease in spread diameter. However, the decrease is not drastic; for instance, tripling the fibres content from 1% to 3% leads to a decrease in the average spread diameter from 290.0 to 287.8 mm or about −0.8%. A higher loss of workability was observed for the fibre content of 4%. Figure 9a,b show pictures of the spread areas for two mixes with a fibre content of 1.0% and 4.0%, respectively. It can be observed that the mixture with the highest fibre content shows some signs of fibre agglomeration within the spread area, while the other mixture had no such effect. In addition, the shape of the spread area assumes a more irregular shape.

It should be stressed that UHPFRC mixes with improved flowability, such as those used in this study, facilitate the casting process but have an increased risk of fibres segregation [30]. This subject will be addressed again in Section 4.4. Based on the authors’ own experience, the spread diameter should not exceed 265 mm to minimise the risk of fibres segregation.

### 4.2. Effect of Specimen Thickness

**Figure 10:**
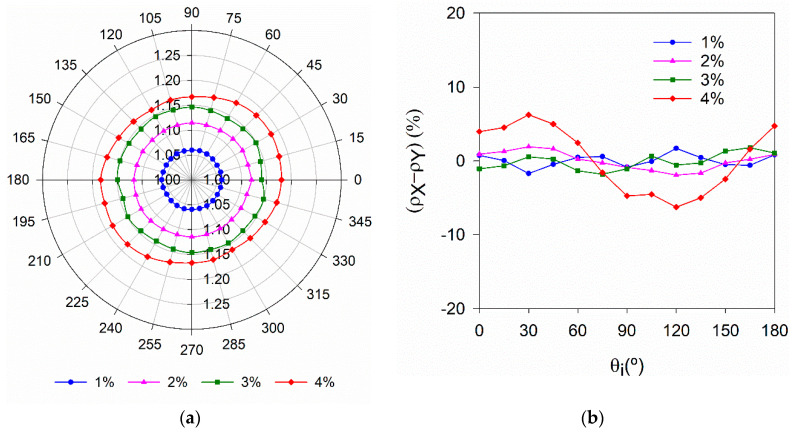
Variation of (**a**) μ_r_ and (**b**) $\left(\rho_{X}-\rho_{Y}\right)$ with the measuring direction (specimens with h_U_ = 100 mm).

Figure 10a shows the variation of the relative magnetic permeability when the probe was aligned in different directions (θ_i_), with θ_i_, ranging from 0° to 180° by steps of 15°. The graphs in Figure 10a were obtained considering μ_r,θi_ = μ_r,(180°-θi)_. Figure 10a clearly shows the increase of μ_r,θi_ with the fibre content. In most specimens, μ_r_ does not change significantly with the measuring direction (data points draw approximately a circle, Figure 10a), confirming a random distribution of fibres. The only exception is the specimen with V_f_ = 4%, where some preferential fibre orientation is observed along θ_i_ = 30° direction (data points draw approximately an ellipse with its major axis aligned with θ_i_ = 30° direction, Figure 10a). This is also clearly indicated by the variation of the orientation indicator in Figure 10b.

**Figure 11:**
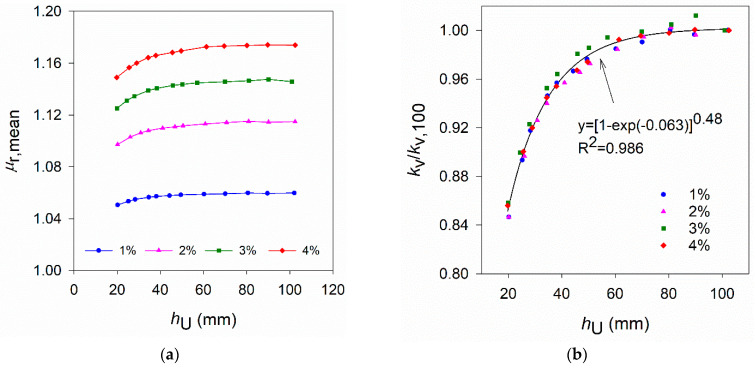
(**a**) Variation of μ_r,mean_ with h_U_; (**b**) Variation of $k_{V}$ normalised to $k_{V,100}$ with h_U_.

Figure 11a shows the variation of the mean relative magnetic permeability for all four specimens as a function of the specimen’s thickness. μ_r,mean_ decreases with the thickness of the specimen, for h_U_ < 70 mm, thus the calibrating constant $k_{V}$ in Equation (3) will also change with h_U_. From Equation (3), for each specimen thickness, $k_{V}$ can be estimated from

(8) $$k_{V}=\frac{V_{\;f}\;}{\left({\;\mu }_{r,\mathrm{mean}}-1\right)}$$

and then, by normalising the results dividing them by the constant obtained for h_U_ = 100 mm ($k_{V,100}$). Figure 11b was obtained. It can be observed that all data points can be fitted by a Chapman-Richards growth function of the type

(9) $$y\left(x\right)=y_{máx}{\left[1-e^{-\mathrm{kx}}\right]}^{p}$$

where $y_{máx}$ = 1.0, and k and p are the fitting parameters. Therefore, when the thickness of the element under analysis does not match the thickness of the specimens used to fit the line in Figure 2b, $k_{V}$ should be corrected using a function similar to the one shown in Figure 11b.

### 4.3. Effect of the Specimen Area

**Figure 12:**
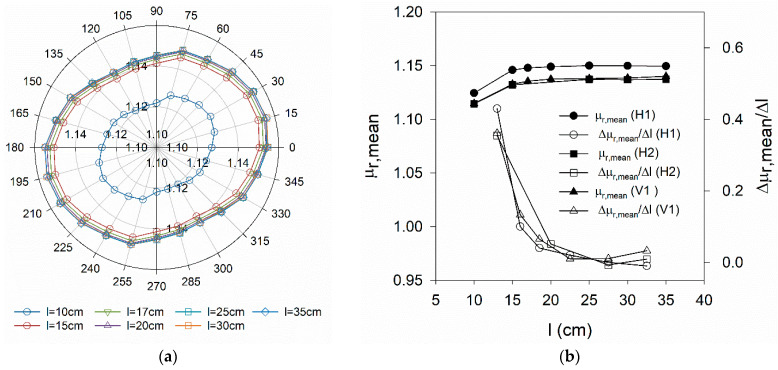
(**a**) Variation of μ_r_ with the measuring direction (V_f_ = 3%) for square specimens with varying size (plate H1)*;* (**b**) Variation of μ_r,mean_ with the specimen size l (plates H1, H2 and V1).

Figure 12a shows the variation of the relative magnetic permeability with the measuring direction of specimens obtained from plate H1. These results reveal a preferential orientation of the fibres in the direction corresponding to θ_i_ = 15°. Figure 12b shows the change in μ_r,mean_ with the reduction of the plates’ size. A similar effect of the specimen’s size was found on the three plates. Figure 11b shows that μ_r,mean_ remains unchanged for specimens with sizes up to 200 mm, decreasing significantly for smaller sizes. Comparing μ_r,mean_ results obtained in specimens of 200 mm size with those of 150 mm (the size of the notched DEWST specimens used in previous campaigns [18,21]), the difference in μ_r,mean_ is relatively small (0.27% in plate H1).

To perform measurements with a probe similar to the one used in this study (Section 2.1), the measuring points over a UHPFRC layer/specimen should be selected so that a minimum area of 200 × 200 mm^2^ surrounding the measuring point exists (the probe is centred on the measuring point). Consequently, NDT measuring points should be at a minimum distance of 100 mm from the sides of the test specimens/elements.

Dog-bone or dumbbell shape specimens are often used in direct tensile tests to characterise the tensile behaviour of UHPFRC [11,12,28]. This type of specimen usually has reduced dimensions in its central part, making it impossible to comply with the recommended minimum distance between the measuring point and the sides of the specimen. Thus, to characterise the fibre content and orientation in the central part of dog-bone or dumbbell shape specimens employing this NDT method, it is recommended to cast a large slab and perform the inductance measurements in the zones of interest (corresponding to the central part of the specimens), and only then cutting the dog-bone/dumbbell shape specimens from the large slab. This procedure was followed in the work of Shen and Brühwiler [12] for scaling the representative tensile response of UHPFRC.

### 4.4. Effect of Surface Roughness

**Figure 13:**
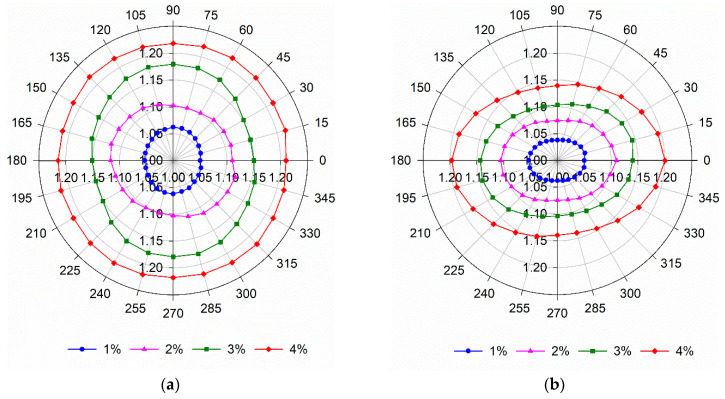
Variation of μ_r_ with the measuring direction for (**a**) HRM_i and (**b**) HOM_i specimens (moulded surface).

**Figure 14:**
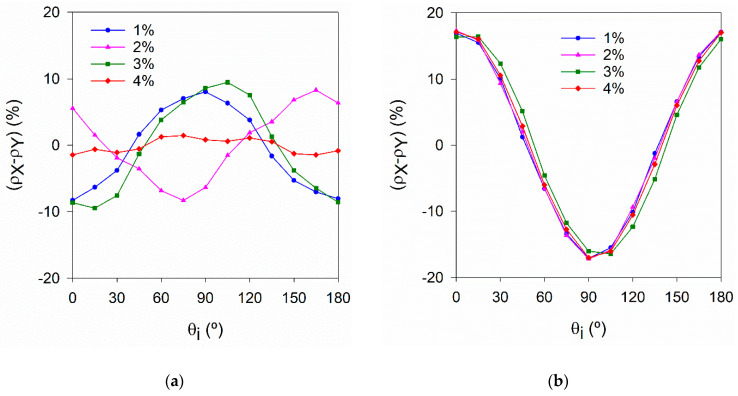
Variation of $\left(\rho_{X}-\rho_{Y}\right)$ with the measuring direction for (**a**) HRM_i and (**b**) HOM_i specimens (moulded surface).

The effectiveness of the orientation set-up [21] used to promote a preferential orientation of the fibres in test series C can be confirmed by comparing the polar plots in Figure 13a,b or the evolution and magnitude of the orientation indicator in Figure 14a,b, corresponding to HRM_i and HOM_i specimens, respectively. A more pronounced orientation of fibres can be found in specimens HOM_i along θ_i_ = 0° (or X direction). Some of the HRM_i specimens also exhibit preferential fibre orientation, originated by the casting conditions, but much less pronounced than in HOM_i specimens. HRM_4% is a good example of a uniform fibre distribution.

**Figure 15:**
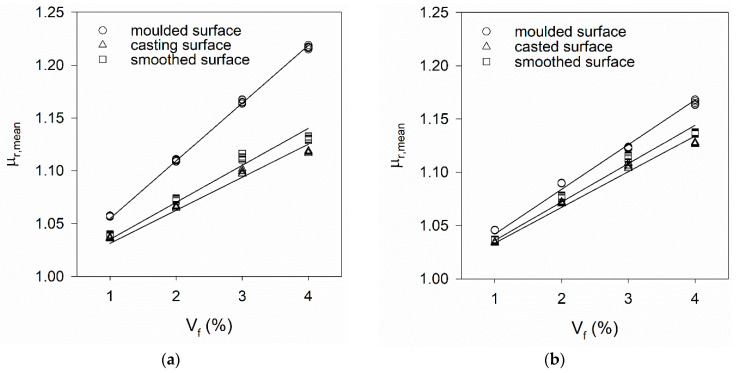
Increase of μ_r,mean_ as a function of V_f_ and corresponding fitting lines for (**a**) HR_i and (**b**) HO_i specimens.

**Figure 16:**
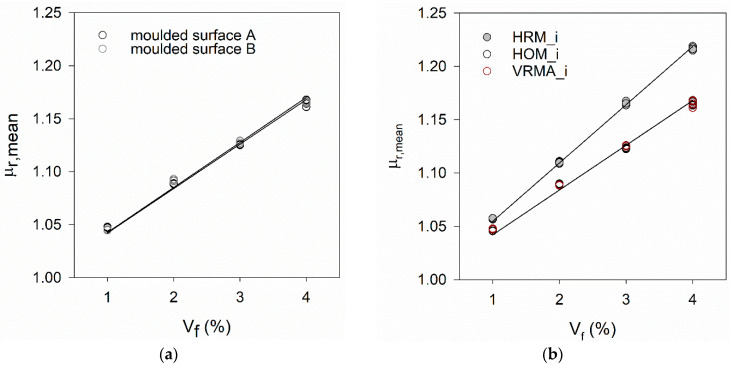
Increase of μ_r,mean_ results as a function of V_f_ and corresponding fitting lines for (**a**) VR_i specimens (**b**) all moulded surfaces.

Figure 15 and Figure 16 show the variation of μ_r,mean_ computed according to Equation (2) as a function of V_f_. These figures include results obtained on surfaces with different types of roughness (moulded, casting, smoothed) and specimens cast both horizontally and vertically. Table 6 summarises the calibrating constant $k_{V}$ and the squared correlation coefficient (*R*^2^) of the fitting lines presented in Figure 15 and Figure 16.

Figure 15 and Figure 16 show the positioning of the mould during casting and the choice of the surface to measure inductance can influence the value of the calibrating constant $k_{V}$. No difference was found in results obtained in both faces of VR_i specimens. However, in both HR_i and HO_i specimens, lower μ_r,mean_ results were obtained in the casting (upper) surface compared to the moulded surface, attributed to the surface roughness and/or fibres segregation (in-depth).

A rough surface does not allow for good contact between the probe and the UHPFRC material, and air gaps are introduced between the probe and the material, significantly reducing the measured inductance. When this occurs, a possible solution is to apply mechanical polishing to the surface to smooth the surface before performing the inductance measurements (see Figure 8). Figure 15 shows μ_r,mean_ results increased after polishing the casting surfaces, proving that a better contact was achieved between the probe and the material. Similar surface treatment was adopted in the work of Shen and Brühwiler [12].

### 4.5. Effect of Fibres Segregation (In-Depth)

**Figure 17:**
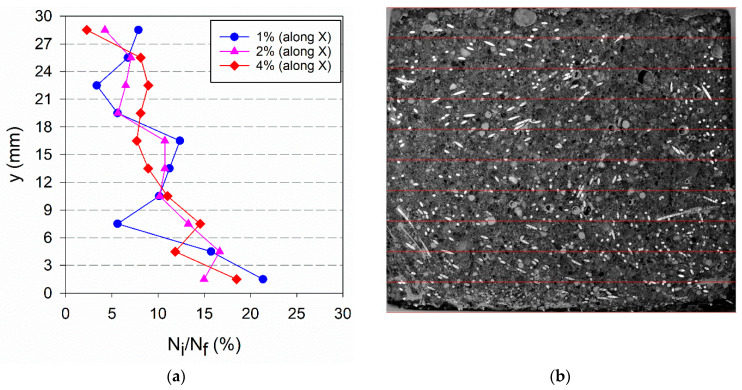
(**a**) Fibre distribution along the plate thickness for HR_i specimens (**b**) image of HR_4% along X-direction.

**Figure 18:**
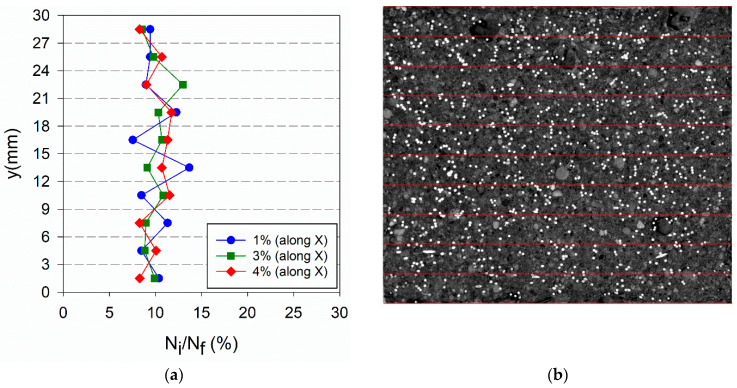
(**a**) Fibre distribution along the plate thickness (X-direction) for HO_i specimens; (**b**) image of HO_4% specimen along X-direction.

When comparing the results obtained on all moulded surfaces (see Figure 16b), from both vertical and horizontal moulds, it can be observed that the fitting lines from VRMA/B_i and HOM_i specimens are overlapped (similar $k_{V}$ results), but are below the fitting line corresponding to HRM_i specimens. In order to understand this difference, further studies were carried out to investigate the occurrence of fibres segregation (in-depth) in HRM_i. For this purpose, small cubic specimens of 30 × 30 × 30 mm^3^ were cut from the centre of plate specimens so that the fibres intersecting a chosen cross-section normal to the X- or Y-axis could be counted (N_f_). The images of each cross-section were subdivided into 3 mm horizontal layers and the number of fibres per layer was manually counted using ImageJ software [31] (see Figure 17b and Figure 18b).

Table 7 summarises the total number of fibres counted in each cross-section. As expected, for the same orientation, N_f_ increases with the fibre content. On the other hand, keeping the fibre content, N_f_ increases when fibres are more aligned with the direction normal to the cross-section.

**Figure 19:**
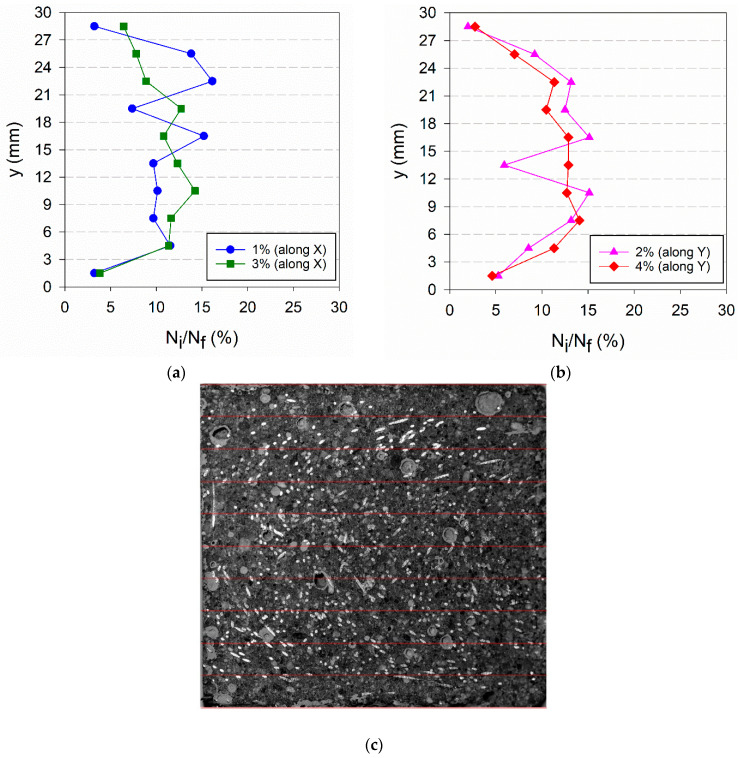
Fibre distribution along the plate thickness for VR_i specimens: (**a**) along X-direction, (**b**) along Y-direction; (**c**) image of VR_4% along X-direction.

The percentage of fibres per layer found is represented in Figure 17a, Figure 18a and Figure 19a, for each type of specimen. In these graphs, y = 0 corresponds to a moulded surface, which is the bottom face for horizontally cast specimens and a lateral face for vertically cast specimens. The fibres distribution in Figure 17 in comparison to Figure 18 confirms the occurrence of fibres segregation in HR_i specimens. More fibres were found in the bottom part (moulded surface) of HR_i specimens compared to the upper part (casting surface), and this explains the higher μ_r,mean_ results of HRM_i in Figure 16b. In-depth fibres segregation in HR_i specimens was promoted by the higher fluidity of the UHPFRC mixes used in this study (see Section 4.1) and, probably, some agitation of the material during casting, which reduces the matrix yield stress causing fibres settlement.

The wall effect is evidenced in the distribution of fibres of VR_i specimens presented in Figure 19. A more symmetric distribution of the fibres along the thickness is observed, and significantly fewer fibres were found in the layers closer to the mould walls compared to the centre of the plate.

**Figure 20:**
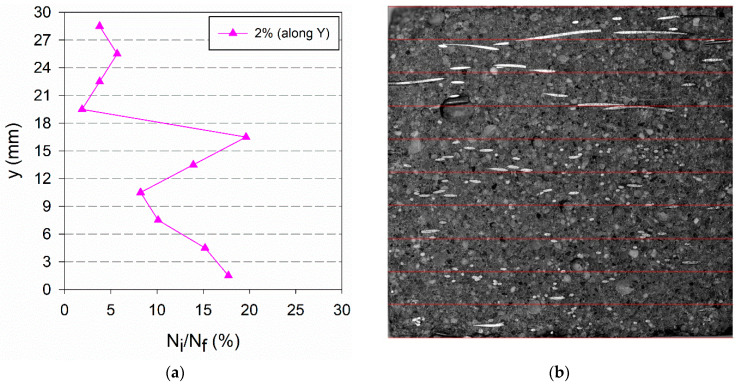
(**a**) Fibre distribution along the plate thickness (Y-direction) for HO_i specimens; (**b**) image of HO_2% along Y-direction.

It is interesting to notice that in HO_i specimens if we analyse a cross-section parallel to the direction of preferential orientation, a much smaller number of fibres is found, and thus the fibres distribution along the plate thickness has a larger scatter, as shown in Figure 20a. This did not occur with other cross-sections normal to the Y-direction, represented in Figure 17a and Figure 18a, because these specimens have a random fibres distribution and thus not differing significantly in X- and Y-directions.

## 5. Conclusions

This paper focused on the effect of key factors for successfully implementing the magnetic NDT method previously developed by the authors [18]. The following conclusions can be drawn from the results presented in this paper:

1. The proportionality constant $k_{V}$ that allows to predict V_f_ from the μ_r,mean_ estimated using the magnetic probe changes with the thickness of the UHPFRC layer (h_U_). A correction function is proposed for $k_{V}$ as a function of h_U_.
2. The magnetic probe used in this study is capable of evaluating the fibre content and fibre orientation in the UHPFRC up to a depth of 70 mm measured from the contact surface. In UHPFRC elements where it is possible to take measurements on two opposite sides the assessed material thickness can be doubled.
3. To perform measurements with a probe similar to the one used in this study, the measuring points (the point at which the probe is centred) are recommended to be at a minimum distance of 100 mm from the sides of the test specimens/elements.
4. The probe measurements should be performed preferably over a moulded and smooth surface since perfect contact between the probe and the UHPFRC material is necessary to obtain representative measurements. Unevenness, for example, in the casting surfaces, should be removed by grinding.
5. The magnetic probe is sensitive to the occurrence of in-depth fibres segregation. Even in thin UHPFRC layers, fibres settlement can occur when casting with more fluid mixtures due to gravity action and/or when the material is agitated during casting.

This study raised the awareness that the thickness, area and roughness of the UHPFRC specimen/element under study, as well as the occurrence of in-depth fibres segregation, can have a significant influence on the inductance results. With the recommendations presented in this paper, one expects to contribute to more efficient use of this NDT method for quality assurance of UHPFRC elements at an industrial scale; being able to provide valuable information on the casting quality (fibres distribution and orientation) and the in-structure tensile strength in any direction.

## Figures and Tables

**Table 1:** Characteristics of the magnetic probe.

U-Shape Ferrite Core				Copper Wire Coil	
Reference	Relative Magnetic Permeability	Length	Cross-Section	Wire Diameter	Number of Turns
Siemens ferrite N47	~2000	189 mm	28 × 30 mm^2^	0.5 mm	1454

**Table 2:** Mix-proportions of UHPFRC employed in this study (kg/m^3^).

UHPFRC	Constituent Materials	V_f_ = 1%	V_f_ = 2%	V_f_ = 3%	V_f_ = 4%
Cementitious matrix	Cement	794.9			
	Silica fume	79.49			
	Limestone filler	311.43			
	Water	145.36			
	Superplasticizer	30			
	Sp/c *	1.51%			
	Sand	993.56	967.26	940.96	914.66
Steel fibres (straight)	l_f_ = 9 mm/d_f_ = 0.175 mm	39.25	78.5	117.5	157
	l_f_ = 12 mm/d_f_ = 0.175 mm	39.25	78.5	117.5	157

(*) percentage of superplasticizer (solid content) by weight of cement.

**Table 3:** NDT testing programme.

Test Series:	A	B	C	
Effect Being Evaluated:	Thickness	Area	Surface Roughness	Fibres Segregation (In-Depth)
specimen’s	cylinder	square plate	square plate	
geometry	h = 100 to 20 mm	l = 350 to 100 mm	l = 200 mm	
	ϕ = 150 mm	h = 30 mm	h = 30 mm	
total fibre content	1%	3%	1%	
	2%		2%	
	3%		3%	
	4%		4%	
number of specimens	4	3	12	
fibres orientation	random	random	random	
			oriented *	
mould position	horizontal	horizontal	horizontal	
		vertical	vertical	
condition of the test surface	moulded	moulded	moulded surface	
			casting surface	
			polished surface	

(*) fibres orientation was achieved by means of an external electromagnetic field.

**Table 4:** Referencing of specimens from test series C.

Mould Position	Fibres Orientation	Condition of the Test Surface	Specimens Reference *	
Horizontal	Random	Moulded surface	HRM_i	HR_i
		Casting surface	HRC_i	
		Smoothed surface	HRS_i	
Horizontal	Oriented	Moulded surface	HOM_i	HO_i
		Casting surface	HOC_i	
		Smoothed surface	HOS_i	
Vertical	Random	Moulded surface A	VRMA_i	VR_i
		Moulded surface B	VRMB_i	

(*) In the specimen reference i refers to the fibre content.

**Table 5:** Slump-flow diameter results (mm).

Test Series	Casting Date	Reference	V_f_ = 1%	V_f_ = 2%	V_f_ = 3%	V_f_ = 4%
A	22 May 2019	--	294.5	295.0	291.5	263.0
C	12 June 2019	HR_i	295.5	289.0	287.5	281.0
	or	HO_i	286.0	287.5	284.0	280.5
	14 June 2019	VR_i	288.5	287.0	288.0	274.0
Average			290.0	289.6	287.8	274.6
Standard deviation			4.9	3.7	3.1	8.4
Coefficient of variation			1.7%	1.3%	1.1%	3.1%

**Table 6:** Calibrating constant $k_{V}$ and *R*^2^ of fitting lines.

Mould Position	Fibres Orientation	Reference	k_V_	*R* ^2^
Horizontal	Random	HRM_i	5.5	0.999
		HRC_i	3.1	0.965
		HRS_i	3.5	0.965
	Oriented	HOM_i	4.2	0.992
		HOC_i	3.4	0.980
		HOS_i	3.6	0.979
Vertical	Random	VRMA_i	4.2	0.991
		VRMB_i	4.3	0.990

**Table 7:** Total number of fibres (N_f_) counted in a square area of 30 mm in size.

Fibre Content	HR_i		HO_i		VR_i	
V_f_	Along X	Along Y	Along X	Along Y	Along X	Along Y
1%	89	--	212	--	217	--
2%	354		--	158		152
3%	--	639	747		713	--
4%	481	--	953	--		583

## References

[1] Graybeal B., Brühwiler E., Kim B.-S., Toutlemonde F., Voo Y.L., Zaghi A. (2020). International Perspective on UHPC in Bridge Engineering. J. Bridge Eng..

[2] Toutlemonde F., Bernadi S., Brugeaud Y., Simon A. (2018). Twenty Years-Long French Experience in UHPFRC Application and Paths Opened from the Completion of the Standards for UHPFRC. https://hal.archives-ouvertes.fr/hal-01955204.

[3] Marek J., Kolisko J., Tej P., Čítek D., Komanec J., Kalný M., Vráblík L. (2019). New UHPFRC bridges in the Czech Republic. IOP Conf. Ser. Mater. Sci. Eng..

[4] López J.Á., Serna P., Navarro-Gregori J., Camacho E. Construction of the U-Shaped Truss Footbridge over the Ovejas Ravine in Alicante. Proceedings of the 2º International Symposium on UHPFRC. Designing and Building with UHPFRC.

[5] Brühwiler E., Denarié E. (2013). Rehabilitation and Strengthening of Concrete Structures Using Ultra-High Performance Fibre Reinforced Concrete. Struct. Eng. Int..

[6] Alberti M.G., Enfedaque A., Galvez J. (2018). A review on the assessment and prediction of the orientation and distribution of fibres for concrete. Compos. Part. B Eng..

[7] Huang H., Gao X., Teng L. (2021). Fiber alignment and its effect on mechanical properties of UHPC: An overview. Constr. Build. Mater..

[8] Pastor F., Hajar Z., Palu P.D. UHPFRC Footbridge in le CANNET des MAURES. Proceedings of the AFGC-ACI-fib-RILEM Int. Symposium on Ultra-High Performance Fibre-Reinforced Concrete, UHPFRC.

[9] Brühwiler E., Bastien-Masse M., Mühlberg H., Houriet B., Fleury B., Cuennet S., Schär P., Boudry F., Maurer M. Strengthening the Chillon viaducts deck slabs with reinforced UHPFRC. Proceedings of the IABSE Conference Geneva 2015 ‘Structural Engineering: Providing Solutions to Global Challenges’.

[10] Bastien-Masse M., Denarié E., Brühwiler E. (2016). Effect of fiber orientation on the in-plane tensile response of UHPFRC reinforcement layers. Cem. Concr. Compos..

[11] Abrishambaf A., Pimentel M., Nunes S. (2017). Influence of fibre orientation on the tensile behaviour of ultra-high performance fibre reinforced cementitious composites. Cem. Concr. Res..

[12] Shen X., Brühwiler E. (2020). Influence of local fiber distribution on tensile behavior of strain hardening UHPFRC using NDT and DIC. Cem. Concr. Res..

[13] Miletić M., Kumar L.M., Arns J.-Y., Agarwal A., Foster S., Arns C., Perić D. (2020). Gradient-based fibre detection method on 3D micro-CT tomographic image for defining fibre orientation bias in ultra-high-performance concrete. Cem. Concr. Res..

[14] Krause M.S., Hausherr J.M., Burgeth B., Herrmann C., Krenkel W. (2010). Determination of the fibre orientation in composites using the structure tensor and local X-ray transform. J. Mater. Sci..

[15] Ferrara L., Faifer M., Toscani S. (2011). A magnetic method for non destructive monitoring of fiber dispersion and orientation in steel fiber reinforced cementitious composites—Part 1: Method calibration. Mater. Struct..

[16] Ferrara L., Faifer M., Muhaxheri M., Toscani S. (2011). A magnetic method for non destructive monitoring of fiber dispersion and orientation in steel fiber reinforced cementitious composites. Part 2: Correlation to tensile fracture toughness. Mater. Struct..

[17] Cavalaro S.H.P., López-Carreño R., Torrents J.M., Aguado A., Juan-García P. (2015). Assessment of fibre content and 3D profile in cylindrical SFRC specimens. Mater. Struct..

[18] Nunes S., Pimentel M., Carvalho A. (2016). Non-destructive assessment of fibre content and orientation in UHPFRC layers based on a magnetic method. Cem. Concr. Compos..

[19] Ozyurt N., Mason T.O., Shah S.P. (2006). Non-destructive monitoring of fiber orientation using AC-IS: An industrial-scale application. Cem. Concr. Res..

[20] Lataste J., Behloul M., Breysse D. (2008). Characterisation of fibres distribution in a steel fibre reinforced concrete with electrical resistivity measurements. NDT E Int..

[21] Nunes S., Pimentel M., Ribeiro F., Milheiro-Oliveira P., Carvalho A. (2017). Estimation of the tensile strength of UHPFRC layers based on non-destructive assessment of the fibre content and orientation. Cem. Concr. Compos..

[22] Li L., Xia J., Chin C., Jones S. (2020). Fibre Distribution Characterization of Ultra-High Performance Fibre-Reinforced Concrete (UHPFRC) Plates using Magnetic Probes. Materials.

[23] Li L., Xia J., Galobardes I. Magnetic probe to test spatial distribution of steel fibres in UHPFRC prisms. Proceedings of the 5th International fib Congress: Better-Smarter-Stronger.

[24] Nunes S., Ribeiro F., Carvalho A., Pimentel M., Brühwiler E., Bastien-Masse M. Non-destructive measurements to evaluate fiber dispersion and content in UHPFRC reinforcement layers. Proceedings of the Multi-Span Large Bridges Conference.

[25] Davis J., Huang Y., Millard S.G., Bungey J. (2003). Determination of Dielectric Properties of Insitu Concrete at Radar Frequencies. Non-Destr. Test. Civ. Eng..

[26] Sine A.G. (2021). Strengthening of Reinforced Concrete Elements with UHPFRC.

[27] Krenchel H. (1975). Fibre spacing and specific fibre surface. Fibre Reinforced Cement and Concrete.

[28] Abrishambaf A., Pimentel M., Nunes S. (2019). A meso-mechanical model to simulate the tensile behaviour of ultra-high performance fibre-reinforced cementitious composites. Compos. Struct..

[29] BIBM, Cembureau, ERMCO, EFCA, EFNARC (2005). The European Guidelines for Self-Compacting Concrete Specification, Production and Use.

[30] Wang R., Gao X., Huang H., Han G. (2017). Influence of rheological properties of cement mortar on steel fiber distribution in UHPC. Constr. Build. Mater..

[31] Rasband W.S. ImageJ.

